# Berberine Protects Human Umbilical Vein Endothelial Cells against LPS-Induced Apoptosis by Blocking JNK-Mediated Signaling

**DOI:** 10.1155/2016/6983956

**Published:** 2016-07-13

**Authors:** Junping Guo, Lijun Wang, Linyao Wang, Senmi Qian, Dayong Zhang, Jie Fang, Jianping Pan

**Affiliations:** ^1^Department of Clinical Medicine, Zhejiang University City College School of Medicine, Hangzhou, Zhejiang 310015, China; ^2^Department of Endocrinology, Zhejiang Provincial People's Hospital, Hangzhou, Zhejiang 310014, China

## Abstract

Endothelial dysfunction is a critical factor during the initiation of atherosclerosis. Berberine has a beneficial effect on endothelial function; however, the underlying mechanisms remain unclear. In this study, we investigated the effects of berberine on lipopolysaccharide- (LPS-) induced apoptosis in human umbilical vein endothelial cells (HUVECs) and the molecular mechanisms mediating the effect. The effects of berberine on LPS-induced cell apoptosis and viability were measured with 5-ethynyl-2′-deoxyuridine staining, flow cytometry, and Cell Counting Kit-8 assays. The expression and/or activation of proapoptotic and antiapoptotic proteins or signaling pathways, including caspase-3, poly(ADP-ribose) polymerase, myeloid cell leukemia-1 (MCL-1), p38 mitogen-activated protein kinase, C-Jun N-terminal kinase (JNK), and extracellular signal-regulated kinase, were determined with western blotting. The malondialdehyde levels, superoxide dismutase (SOD) activity, and production of proinflammatory cytokines were measured with enzyme-linked immunosorbent assays. The results demonstrated that berberine pretreatment protected HUVECs from LPS-induced apoptosis, attenuated LPS-induced injury, inhibited LPS-induced JNK phosphorylation, increased MCL-1 expression and SOD activity, and decreased proinflammatory cytokine production. The effects of berberine on LPS-treated HUVECs were prevented by SP600125, a JNK-specific inhibitor. Thus, berberine might be a potential candidate in the treatment of endothelial cell injury-related vascular diseases.

## 1. Introduction

Endothelial cells (ECs), which act as a selective barrier between tissue and blood, play a potential role in the control of inflammatory responses, immunity, and homeostasis [1–3]. In order to maintain normal organ function and vascular homeostasis, the integrity of the endothelial lining is critical [4, 5]. EC dysfunction and/or injury can disrupt the integrity of the endothelial lining and subsequently lead to vascular disease. EC dysfunction and/or injury, which are commonly mediated by lipopolysaccharide (LPS), are complications of sepsis, which is considered the major cause of several diseases, including diabetes mellitus [6, 7], atherosclerosis [8], and thrombosis [9]. Therefore, agents that protect the vascular endothelium from injury and/or dysfunction are thought to reduce the incidence of cardiovascular disease.

Because LPS is an integral part of the outer membrane of Gram-negative bacteria, it is considered a trigger of EC injury and its associated syndromes. In vitro, LPS stimulation alters multiple EC functions, including viability, apoptosis, malondialdehyde (MDA) release, and tumor necrosis factor- (TNF-)*α* and interleukin- (IL-) 6 synthesis [10, 11].

Increasing evidence suggests that oxidative stress that is induced by LPS stimulation can lead to apoptosis or death of ECs [12, 13]. In ECs, oxidative stress triggers various signal transduction pathways that are related to cell survival and apoptosis, induces damage to cell membranes [14] and DNA structure, and affects members of the mitogen-activated protein (MAP) kinase (MAPK) family and the cell biological processes that are regulated by MAP kinase, such as cell apoptosis, differentiation, and growth. Previous studies have shown that three MAPK subfamilies are activated in response to LPS stimulation [15], including c-Jun N-terminal kinase (JNK), p38 MAP kinase (p38), and extracellular signal-regulated kinase 1/2 (Erk1/2). The activation of these MAPK pathways induces proapoptotic or antiapoptotic reactions [16], depending on the cell type [17–20].

Berberine, which is an isoquinoline alkaloid derivative that is extracted from* Rhizoma Coptidis* (Huang Lian, a traditional Chinese medicine), exhibits various pharmacological activities, including antiproliferative [21], anti-inflammatory [22], antihyperlipidemic, antidiabetic [23], antidiarrheal, antimicrobial [24], and antioxidative [25] actions. Therefore, we hypothesized that berberine treatment would protect human umbilical vein endothelial cells (HUVECs) against LPS-induced injury.

In the present study, an in vitro model of LPS-induced HUVEC injury was used to evaluate the cytoprotective effects of berberine on LPS-induced HUVEC injury. Our results demonstrated that berberine protected HUVECs from LPS-induced injury by blocking activation of the JNK pathway and subsequently promoting the expression of the antiapoptotic protein myeloid cell leukemia-1 (MCL-1).

## 2. Materials and Methods

### 2.1. Cell Culture and Treatment

HUVECs were isolated from the vein of a normal human umbilical cord and cultured in DMEM-F12 medium supplemented with 10% fetal bovine serum, 2 mM l-glutamine, 5 U/mL heparin, 100 U/mL penicillin, 10 *μ*g/mL streptomycin, and 50 *μ*g/mL EC growth supplement, as previously described [26]. The cells were cultured and grown in 100 mm dishes under humidified 5% CO_2_ conditions. The medium was refreshed every 2-3 days at cell confluence. The cells were used in the following experiments at passages 3 to 8. For all experiments, the cells were seeded at a concentration of 1 × 10^5^ cells/mL. The cells were pretreated with culture medium containing different concentrations of berberine (1.25, 2.5, or 5 *μ*M) after the cells reached subconfluence. After the 24 h pretreatment, the cells were washed three times with phosphate-buffered saline (PBS; pH 7.4) and exposed to LPS (5 *μ*g/mL) in culture medium at 37°C for 24 h.

### 2.2. Cell Viability Assay

To evaluate the viability of the HUVECs subjected to different treatments, Cell Counting Kit- (CCK-) 8 reduction assay kits (Beyotime Institute of Biotechnology, Jiangsu, China) were employed according to the manufacturer's protocols. Briefly, the cells were incubated with the indicated concentrations of berberine. After the 24 h incubation, the cells were washed and cultured with 5 *μ*g/mL of LPS for another 24 h in flat-bottom 96-well plates at a density of 2.0 × 10^4^ cells per well. Next, 20 *μ*L of the CCK-8 reagent was added after 24 h LPS stimulation, and the cells were cultured for an additional 4 h. A M680 microplate reader (Bio-Rad, Marnes-la-Coquette, France) was used to determine the optical density at 450 nm.

### 2.3. 5-Ethynyl-2′-deoxyuridine (EdU) Incorporation and Fluorescent Staining of Cells with Hoechst 33258

The cells were cultured on glass coverslips in medium, and the EdU reagent (final concentration, 50 *μ*M) was added to the culture medium. After 24 h in culture, the cells were fixed with a standard formaldehyde fixation protocol. The cells were then stained with Hoechst 33442 (10 *μ*g/mL) under dark conditions at room temperature for 10 min to determine the nuclear morphology of the apoptotic cells. The cells were washed with PBS and observed with fluorescent microscopy (excitation, 340 nm; emission, 460 nm; AX80, Olympus Corporation, Tokyo, Japan).

### 2.4. Detection of Cell Apoptosis with Flow Cytometry

The apoptosis in the HUVECs that was induced by LPS was measured quantitatively with Annexin V and propidium iodide (PI) staining in a flow cytometric analysis [27]. Briefly, the cells were pretreated with different concentrations of berberine at 37°C for 24 h and then stimulated with LPS (5 *μ*g/mL) for an additional 24 h. After another 24 h, the cells were collected and washed three times with cold PBS. Next, the cells were incubated and stained with Annexin V-fluorescein isothiocyanate (10 *μ*L 20 *μ*g/mL) at 4°C under dark conditions for 30 min. After three washes with binding buffer, the cells were stained with 5 *μ*L of PI for 10 min. The cells were kept on ice without exposure to light before the flow cytometry analysis.

### 2.5. Detection of MDA and Superoxide Dismutase (SOD)

The HUVECs were pretreated with different concentrations of berberine for 24 h and then stimulated with LPS for another 24 h. The cells were lysed with cell lysis buffer containing 1 mM phenylmethylsulfonyl fluoride, 1 *μ*g/mL leupeptin, 1 mM Na_3_VO_4_, 1 mM glycerophosphate, 2.5 mM sodium pyrophosphate, 1% Triton X-100, 1 mM ethylene glycol tetraacetic acid, 1 mM ethylenediaminetetraacetic acid, 150 mM NaCl, and 20 mM Tris-HCl. Next, the cell lysates were collected and used to determine the levels of MDA and activity of SOD. The protein levels in the lysates were measured with a bicinchoninic acid protein assay kit (Bio-Rad Laboratories, Inc., Hercules, CA, USA). The MDA levels and SOD activity were determined with commercially available kits. The MDA levels, which were based on the formation of a stable chromophoric product from a reaction with thiobarbituric acid, were measured at a wavelength of 532 nm. The MDA values were expressed as nM/mg protein. The SOD activity was based on its ability to inhibit the oxidation of hydroxylamine by the xanthine-xanthine oxidase system. The SOD activity values were expressed as the amount that reduced the absorbance at 550 nm by 50%.

### 2.6. Enzyme-Linked Immunosorbent Assays

The production of inflammatory cytokines, including TNF-*α* and IL-6, in the culture supernatants was determined with enzyme-linked immunosorbent assay kits (R&D Systems, Inc., Minneapolis, MN, USA, and eBioscience, Inc., San Diego, CA, USA, resp.) according to the manufacturers' instructions.

### 2.7. Western Blotting

The cells were pretreated with different concentrations of berberine for 24 h and then stimulated with LPS for another 24 h. After the LPS stimulation, the cells were collected and washed three times with cold PBS. The cells were then lysed with a cell lysis buffer containing 1 mM phenylmethylsulfonyl fluoride, 1 *μ*g/mL leupeptin, 1 mM Na_3_VO_4_, 1 mM glycerophosphate, 2.5 mM sodium pyrophosphate, 1% Triton X-100, 1 mM ethylene glycol tetraacetic acid, 1 mM ethylenediaminetetraacetic acid, 150 mM NaCl, and 20 mM Tris-HCl. The protein concentrations in the cell lysates were determined with a bicinchoninic acid protein kit (Bio-Rad Laboratories, Inc.). Equal amounts of protein were then separated with sodium dodecyl sulfate-polyacrylamide gel electrophoresis. The proteins were electrophoretically transferred to polyvinylidene fluoride membranes at 300 mA for 90 min at a low temperature. The membranes were blocked with 5% skim milk for 1 h at room temperature. After three washes, the membranes were incubated with the indicated primary antibodies at room temperature for 2 h or at 4°C overnight. The membranes were washed three times with PBS containing Tween-20 and incubated with anti-mouse or anti-rabbit IgG secondary antibodies at room temperature for 1 h. After the 1 h incubation, the membranes were washed three times with PBS containing Tween-20, and an enhanced chemiluminescence kit (GE Healthcare Bio-Sciences, Pittsburgh, PA, USA) was used to visualize the protein bands on the membranes. The following antibodies (all from Abcam plc, Cambridge, UK) were used: rabbit anti-human caspase-3 (ab4051), rabbit anti-human poly(ADP-ribose) polymerase (PARP, ab6079), mouse anti-human MCL-1 (ab114016), rabbit anti-human p38 (ab170099), rabbit anti-human phospho-p38 (ab178867), rabbit anti-human Erk1/2 (ab184699), rabbit anti-human phospho-Erk1/2 (ab200807), rabbit anti-human JNK (ab179461), rabbit anti-human phospho-JNK (ab4821), mouse anti-human *β*-actin (ab8226), goat anti-mouse IgG (horseradish peroxidase, ab6789), and goat anti-rabbit IgG (horseradish peroxidase, ab6721). The ImageJ V1.46r software was used to analyze the results of Western blot assay.

### 2.8. Statistical Analysis

The statistical analyses were performed with the SPSS 10.0 package (IBM Corporation, Armonk, NY, USA). The statistical comparisons were performed with analysis of variance (ANOVA) tests, which were followed by Fisher's protected least significance difference (PLSD) tests. All of the values are expressed as mean ± standard error of the mean from at least three independent experiments. The differences between the groups with *P* values less than 0.05 were considered significant. 

## 3. Results

### 3.1. Berberine Increased the Viability of LPS-Treated HUVECs

**Figure 1:**
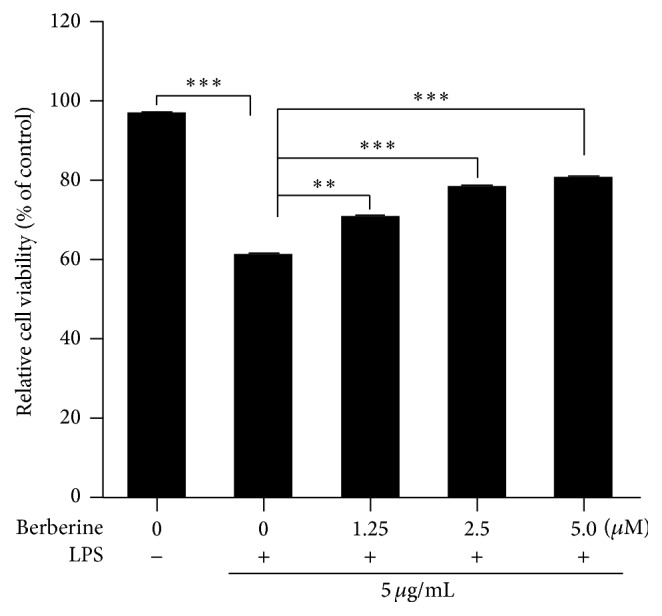
Effects of berberine on cell viability in the human umbilical vein endothelial cells (HUVECs). The HUVECs were incubated with various concentrations of berberine for 24 h. The cells were then washed with fresh medium three times and cultured with lipopolysaccharide (LPS; 5 *μ*g/mL) for another 24 h. After stimulation with LPS, cell viability was measured with the Cell Counting Kit- (CCK-) 8 method. The values represent the mean ± standard error of the mean (SEM; *n* = 3 independent experiments). ^*∗∗*^ *P* < 0.01; ^*∗∗∗*^ *P* < 0.001.

Because cell viability is the most direct indicator of cell state, CCK-8 cell viability kits were used to evaluate the effects of berberine on the viability of LPS-induced HUVECs. As shown in Figure 1, LPS induction significantly decreased cell viability in the HUVECs compared with the untreated control group. However, the decrease in cell viability that was induced by LPS was dose dependently attenuated by berberine. These results indicated that berberine was potentially protective against in vitro LPS-induced HUVEC injury.

### 3.2. Berberine Decreased the Levels of MDA and Increased the Activity of SOD

**Figure 2:**
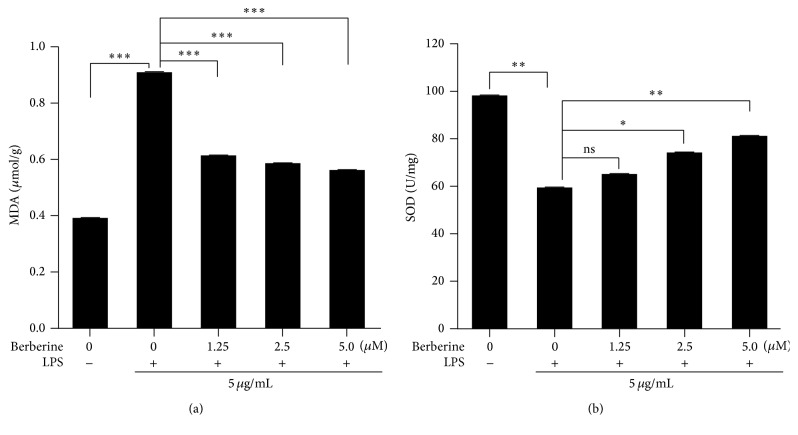
Effects of berberine on the release of malondialdehyde (MDA) and the activity of superoxide dismutase (SOD) in the LPS-treated HUVECs. (a) The levels of MDA in the HUVECs subjected to different treatments. (b) The activity of SOD in the HUVECs subjected to different treatments. The values represent the mean ± SEM (*n* = 3 independent experiments). ^*∗*^ *P* < 0.05; ^*∗∗*^ *P* < 0.01; ^*∗∗∗*^ *P* < 0.001.

In order to investigate the mechanisms by which berberine protected HUVECs from LPS-induced injury, the levels of oxidative stress in the HUVECs that were stimulated by LPS were determined. As shown in Figure 2(a), LPS induction significantly increased the levels of MDA in the HUVECs. However, this LPS-induced increase in the MDA levels in the HUVECs was decreased by berberine treatment (Figure 2(a)). In addition, we measured SOD activity in the HUVECs that were subjected to different treatments, as shown in Figure 2(b). Compared with the untreated control group, SOD activity was inhibited by the LPS treatment, and the LPS inhibition was significantly prevented by treatment with different concentrations of berberine. These observations demonstrated that berberine protected the HUVECs from LPS-induced oxidative injury.

### 3.3. Berberine Decreased the Levels of Inflammatory Cytokines in LPS-Induced HUVEC Injury

**Figure 3:**
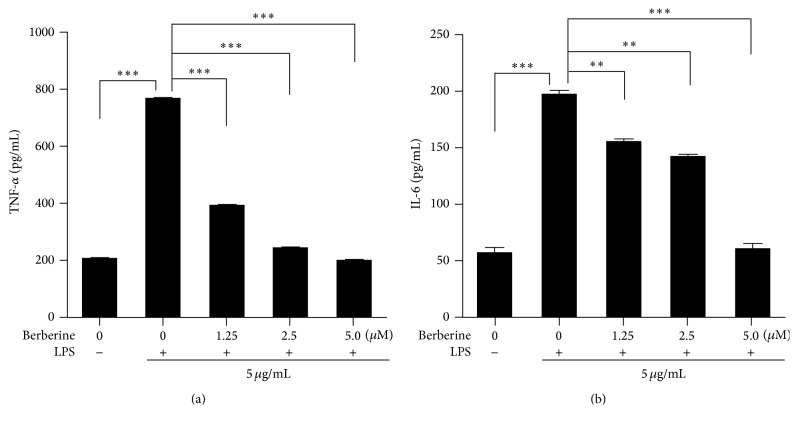
Effects of berberine on the levels of LPS-induced proinflammatory cytokines in the HUVECs. (a) The levels of tumor necrosis factor- (TNF-)*α* in the HUVECs subjected to different treatments. (b) The levels of interleukin- (IL-) 6 in the HUVECs subjected to different treatments. The values represent the mean ± SEM (*n* = 3 independent experiments). ^*∗∗*^ *P* < 0.01; ^*∗∗∗*^ *P* < 0.001.

In order to determine whether berberine suppressed the inflammatory responses in the HUVECs that were induced by LPS stimulation, we pretreated the HUVECs with various concentrations of berberine for 24 h. The cells were then cultured with LPS (5 *μ*g/mL) for another 24 h. The levels of inflammatory cytokines, including TNF-*α* and IL-6, were measured in the culture supernatants. As shown in Figure 3, LPS induction significantly increased the levels of TNF-*α* (Figure 3(a)) and IL-6 (Figure 3(b)) in the HUVEC culture supernatants. However, berberine administration inhibited the LPS stimulation-induced increase in the levels of these two inflammatory cytokines in the HUVECs. These results indicated that berberine had potential anti-inflammatory properties in the HUVEC model of LPS-induced injury.

### 3.4. Berberine Protected the HUVECs from LPS-Induced Apoptosis

**Figure 4:**
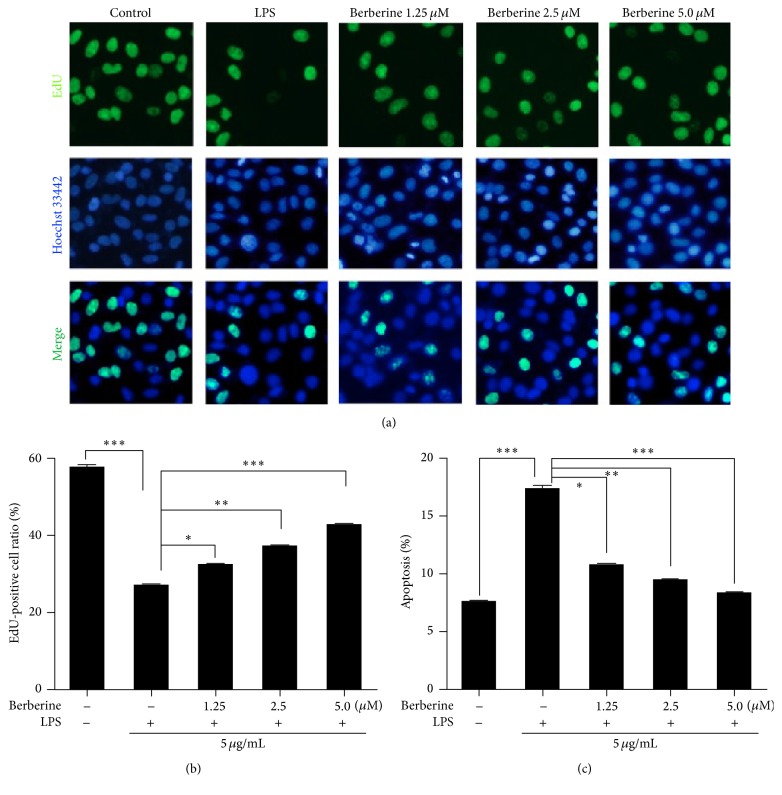
Effects of berberine on LPS-induced apoptosis in the HUVECs. The cells were pretreated with various concentrations of berberine for 24 h and then exposed to LPS for another 24 h. Apoptosis was determined in the cells that were subjected to different treatments with the DNA-binding 5-ethynyl-2′-deoxyuridine (EdU)/Hoechst 33442 double staining and Annexin V/propidium iodide (PI) double staining. (a) Fluorescent micrographs of nuclei in HUVECs subjected to different treatments. (b) The percentage of apoptotic cells in cells subjected to different treatments. (c) Apoptosis was determined in the cells subjected to different treatments with Annexin V/PI double staining. The values represent the mean ± SEM (*n* = 3 independent experiments). ^*∗*^ *P* < 0.05; ^*∗∗*^ *P* < 0.01; ^*∗∗∗*^ *P* < 0.001.

The results described above suggested that berberine treatment protected the cells from the LPS-induced loss of cell viability (Figure 1). To further clarify the antiapoptotic and cytoprotective mechanisms of berberine on LPS-induced HUVECs, the HUVECs were pretreated with various concentrations of berberine for 24 h and then stimulated with LPS. After the LPS stimulation, cell apoptosis was analyzed with Hoechst 33442/EdU staining and Annexin V/PI double staining. According to a previous report [28], cells that exhibit nuclear fragmentation, intense fluorescence, chromatin condensation, and reduced nuclear size are considered apoptotic. As shown in Figures 4(a) and 4(b), significant nuclear fragmentation was observed in the LPS-induced cells compared with the control cells. These apoptotic changes in the nucleus were dramatically rescued by pretreatment with various concentrations of berberine.

To confirm the antiapoptotic effects of berberine on the LPS-induced HUVECs, we assessed the levels of apoptosis in cells that underwent different treatments with flow cytometry and the Annexin V/PI double-staining system. As shown in Figure 4(c), an increase in the percentage of apoptotic cells was observed in the LPS-treated group compared with the control group. However, berberine pretreatment decreased the percentage of apoptotic cells after LPS exposure. This antiapoptotic effect was concentration-dependent.

### 3.5. Berberine Protected HUVECs from LPS-Induced Injury by Blocking JNK Phosphorylation

**Figure 5:**
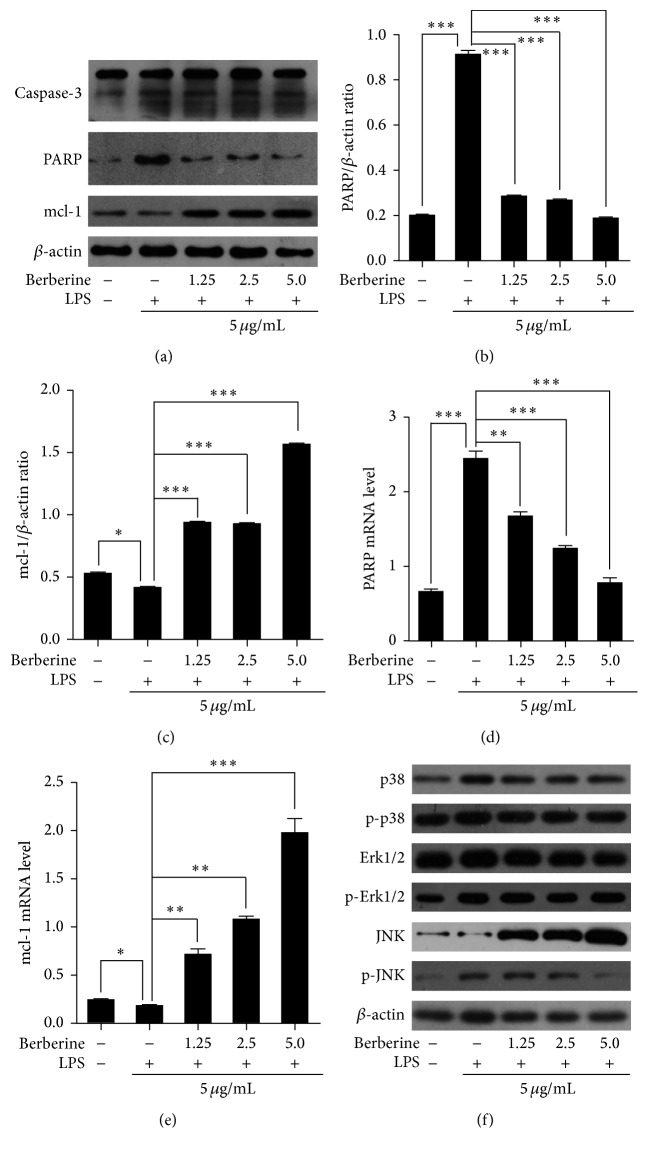
Effects of berberine on the levels of expression of apoptotic markers, phospho-p38 mitogen-activated protein kinase (p38), phosphoextracellular signal-regulated kinase 1/2 (Erk1/2), and phospho-C-Jun N-terminal kinase (JNK) in HUVECs in response to LPS. The cells were pretreated with various concentrations of berberine for 24 h and then incubated with LPS (5 *μ*g/mL) for another 24 h. After LPS treatment, the cells were harvested, and the levels of caspase-3, poly(ADP-ribose) polymerase (PARP), and myeloid cell leukemia- (MCL-) 1 and the phosphorylation of p38, Erk1/2, and JNK were measured with western blotting and qPCR methods. (a) Representative of the protein levels of caspase-3, PARP, and MCL-1 in the cells subjected to different treatments; (b-c) representative of the quantification of the protein levels of PARP (b) and MCL-1 (c) in the cells subjected to different treatments; (d-e) representative of the mRNA levels of PARP (d) and MCL-1 (e) in the cells subjected to different treatments; (f) representative of the phosphorylation of p38, Erk1/2, and JNK in the cells subjected to different treatments. One representative experiment of three is shown. The values represent the mean ± SEM (*n* = 3 independent experiments). ^*∗*^ *P* < 0.05; ^*∗∗*^ *P* < 0.01; ^*∗∗∗*^ *P* < 0.001.

To elucidate the detailed mechanisms underlying the protective effects of berberine on the LPS-induced HUVECs, several apoptosis-related molecules were analyzed with western blotting and qPCR methods. As shown in Figures 5(a)–5(e), berberine treatment significantly increased the levels of MCL-1, which is an antiapoptotic protein, and decreased the levels of the proapoptotic protein PARP in LPS-treated HUVECs compared with the levels in the untreated cells. However, the levels of caspase-3 remained unchanged among the groups (Figure 5(a)). These results indicated that berberine inhibited the LPS-induced apoptosis in HUVECs by affecting the expression of MCL-1 and PARP.

Activation of the JNK and MAPK pathways might be involved in apoptosis [29–31]. In order to clarify whether activation of the JNK and MAPK pathways was involved in the berberine inhibition of the LPS-induced apoptosis of the HUVECs, we next examined the activation of p38, Erk1/2, and JNK in the LPS-treated HUVECs. As shown in Figure 5(f), no significant changes were observed in p38 or Erk1/2 activation in the HUVECs that were treated with different concentrations of berberine. However, a dose-dependent decrease in JNK phosphorylation was observed after berberine treatment. These results suggested that berberine exerted its antiapoptotic effects by blocking activation of the JNK signaling pathway.

**Figure 6:**
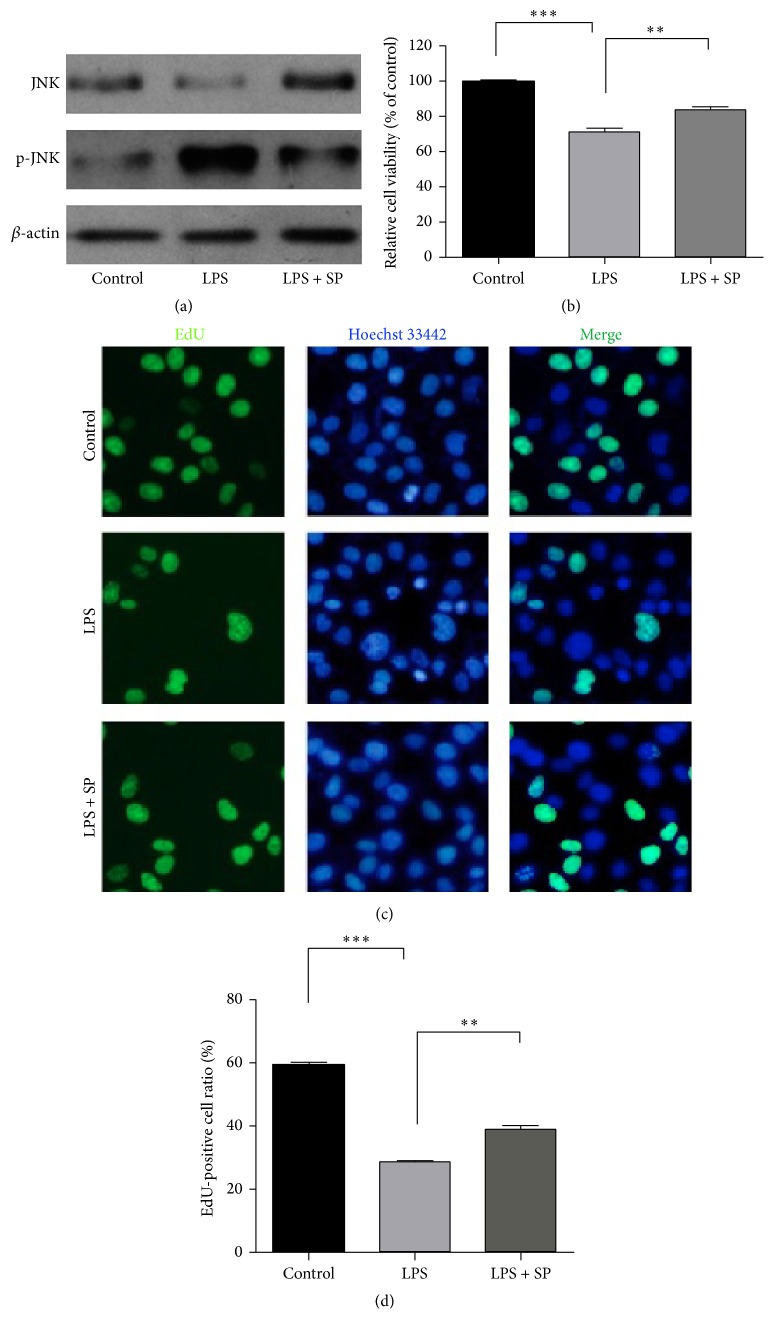
Role of the JNK pathway in LPS-induced HUVEC injury. (a) The expression and phosphorylation of JNK in HUVECs subjected to different treatments. (b) The cell viability of HUVECs subjected to different treatments. (c), (d) The apoptosis of HUVECs subjected to different treatments. The values represent the mean ± SEM (*n* = 3 independent experiments). ^*∗∗*^ *P* < 0.01; ^*∗∗∗*^ *P* < 0.001.

In order to confirm the role of the JNK signaling pathway in LPS-induced HUVEC injury, SP600125 was employed to block the activation of JNK during the HUVEC injury that was induced by LPS treatment. As shown in Figure 6(a), LPS induction significantly increased the phosphorylation of JNK compared with that in the control group, and this increase was dramatically reduced by treatment with SP600125. In accordance with the JNK phosphorylation results, the decreased cell viability that was induced by LPS was significantly restored with SP600125 treatment (Figure 6(b)). In addition, SP600125 administration protected the cells against LPS-induced apoptosis (Figures 6(c) and 6(d)). These results indicated that activation of the JNK pathway played a potentially regulatory role during LPS-induced HUVEC injury and that the inhibition of JNK phosphorylation restored the cell viability of the LPS-treated HUVECs.

**Figure 7:**
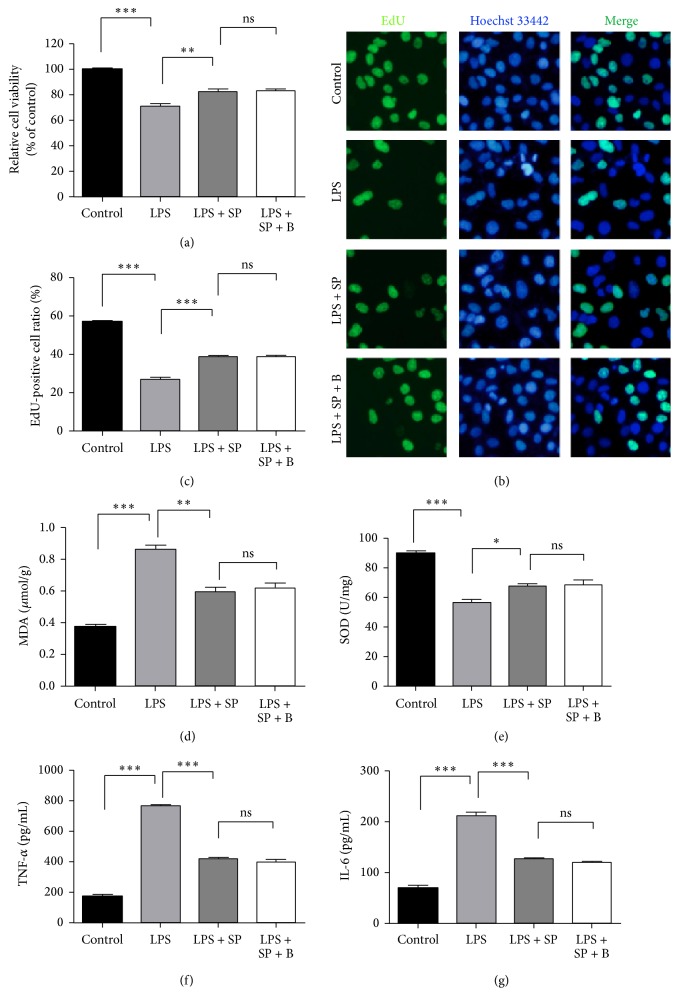
Berberine protects the HUVECs against LPS-induced injury by blocking the JNK signaling pathway. (a) Cell viability in HUVECs subjected to different treatments. (b) Micrographs of the fluorescence of nuclei in HUVECs subjected to different treatments. (c) The percentage of apoptotic cells in the cells subjected to different treatments. (d) The levels of MDA in HUVECs subjected to different treatments. (e) The activity of SOD in HUVECs subjected to different treatments. (f) The levels of TNF-*α* in HUVECs subjected to different treatments. (g) The levels of IL-6 in HUVECs subjected to different treatments. The values represent the mean ± SEM (*n* = 3 independent experiments). ^*∗*^ *P* < 0.05; ^*∗∗*^ *P* < 0.01; ^*∗∗∗*^ *P* < 0.001.

These results clearly demonstrated that activation of the JNK pathway was involved in LPS-induced HUVEC injury and that blockade of the activation of the JNK pathway with a JNK inhibitor effectively suppressed LPS-induced injury in the HUVECs. Therefore, the effects of a JNK inhibitor on the berberine regulation of cell viability and the levels of MDA, SOD, and the proinflammatory cytokines were examined in LPS-treated HUVECs. As shown in Figure 7, all of the berberine-exerted regulatory effects on the LPS-treated HUVECs, including the cytoprotective effects (Figures 7(a), 7(b), and 7(c)), MDA production (Figure 7(d)), SOD activity (Figure 7(e)), and production of inflammatory cytokines (Figures 7(f) and 7(g)), were prevented when the JNK pathway was blocked by SP600125. These results further confirmed that berberine protected the HUVECs against LPS-induced injury by blocking the LPS-activated JNK signaling pathway.

## 4. Discussion

A previous study showed that the apoptosis of ECs plays an important role in various vascular endothelial diseases. Similar to LPS stimulation, EC apoptosis could be induced or increased in vitro in response to inflammation. The results of the present study showed that berberine strongly inhibited the apoptosis and increased levels of MDA and inflammatory cytokines that were induced by in vitro LPS treatment in HUVECs and concomitantly increased the activity of SOD. Berberine treatment also significantly suppressed the phosphorylation of JNK in the LPS-treated HUVECs. Furthermore, JNK inhibition by SP600125 abrogated the protective effects of berberine on the LPS-induced injury of the HUVECs. Taken together, these results demonstrated that berberine exerted its cytoprotective effects on the LPS-induced injury of the HUVECs by blocking, at least in part, activation of the JNK signaling pathway.

Oxidative stress is considered a critical pathogenic factor in the process of EC injury under inflammatory conditions, including LPS stimulation [32]. The levels of oxidative stress in vivo or in vitro are associated with the severity of EC injury [33]. Therefore, several antioxidant agents, such as Vitamin C [34], propofol [35], and sphingosine-1-phosphate [36], exhibit protective effects on EC injury from excessive oxidative stress. In injured ECs, the antioxidant enzyme SOD is considered an effective antioxidant defense, and the levels of MDA reflect the severity of the cell damage that is induced by oxidative stress. The results of the present study showed that berberine had strong antioxidative effects on LPS-induced HUVEC injury. Berberine pretreatment inhibited the increases in the levels of MDA and activity of SOD in LPS-treated HUVECs, which suggested that the protective effects of berberine might be related to its antioxidative properties.

Excessive oxidative stress can directly or indirectly lead to cell death, apoptosis, or mitochondrial dysfunction [37]. The results of the present experiments showed that apoptosis was significantly induced by LPS treatment in the HUVECs [38]. However, berberine treatment strongly inhibited the LPS-induced apoptosis and increased cell viability. In addition, berberine treatment enhanced the expression of the antiapoptotic protein MCL-1 and inhibited the expression of the proapoptotic protein PARP. Many studies have indicated that the activation of MAPKs, including p38, Erk1/2, and JNK, is involved in the regulation of cell survival and apoptosis in response to oxidative stress and inflammatory stimulation. In order to elucidate the detailed mechanisms of the protective effects of berberine against LPS-induced apoptosis in HUVECs, the activation of different MAPKs was examined in HUVECs subjected to different treatments. Western blot analyses showed that the phosphorylation of JNK that was induced by LPS treatment was dose dependently inhibited by berberine and that this inhibitory effect of berberine on JNK phosphorylation was abrogated when SP600125 was added to the culture. Furthermore, no significant changes in p38 or Erk1/2 activation were observed after berberine treatment. These results suggested that berberine exerted its antiapoptotic potency by blocking activation of the JNK pathway.

Taken together, the results of the present study showed that berberine protected the HUVECs from LPS-induced injury through its antiapoptotic and antioxidative properties. Berberine ameliorated the LPS-induced apoptosis in the HUVECs by promoting the expression of the antiapoptotic protein MCL-1 and inhibiting the expression of the proapoptotic protein PARP and the phosphorylation of JNK. These findings suggested that berberine is a potential candidate in the treatment of EC injury-related vascular diseases.

## Abbreviations

HUVECs: Human umbilical vein endothelial cells
LPS: Lipopolysaccharide
EdU: 5-Ethynyl-2′-deoxyuridine
PARP: Poly(ADP-ribose) polymerase
MCL-1: Myeloid cell leukemia-1
SOD: Superoxide dismutase
MDA: Malondialdehyde
MAPK: Mitogen-activated protein kinase
JNK: c-Jun N-terminal kinase
p38: p38 mitogen-activated protein kinase
Erk1/2: Extracellular signal-regulated kinase 1/2
TNF-*α*: Tumor necrosis factor-*α*
IL-6: Interleukin-6
ECs: Endothelial cells
PBS: Phosphate-buffered saline
CCK-8: Cell Counting Kit-8
PI: Propidium iodide.
